# Clinical practice guidelines in fibromyalgia. Physiotherapists’ adherence in Denmark: a cross-sectional web-based survey study

**DOI:** 10.1007/s00296-026-06084-6

**Published:** 2026-02-18

**Authors:** J. E. Ferrández-Gómez, M. Gacto-Sánchez, K. Oertel Frederiksen, A. Baño-Alcaraz

**Affiliations:** 1 Center for Translational Research in Physiotherapy, Pathology and Surgery Department, School of Medicine, University of Miguel Hernández de Elche, Ctra. Nacional N-332 Km 8.7, 03550 San Juan de Alicante, Spain; 2 Faculty of Physiotherapy, Occupational Therapy and Podiatry, UCAM Catholic University of Murcia, Murcia, Spain; 3 Department of Physical Therapy, Faculty of Medicine, CEIR Campus Mare Nostrum (CMN), University of Murcia, Instituto Murciano de Investigación Biosanitaria-Virgen de la Arrixaca (IMIB-Arrixaca), El Palmar, Murcia, Spain; 4 Department of Physiotherapy, University College South Denmark, Degnevej 16, 6705 Esbjerg, Denmark

**Keywords:** Surveys and questionnaires, Fibromyalgia, Clinical practice guidelines, Therapeutic strategy, Physiotherapy, Adherence, Chronic pain, Denmark

## Abstract

Fibromyalgia is a widespread chronic condition. Although Clinical Practice Guidelines for fibromyalgia currently exist, their application in diagnostic and therapeutic settings faces significant implementation challenges across healthcare systems. This has led to a persistent gap between knowledge and clinical practice, a phenomenon yet to be explored in the Danish context. A cross-sectional study was conducted to investigate the level of knowledge and adherence to Clinical Practice Guidelines for fibromyalgia among Danish physical therapists, throughout a questionnaire consisting in the selection of the elements that they would consider for the adequate assessment, treatment, and expected duration of the therapeutic approach, to classify professionals as adherent, partially adherent, or non-adherent based on their selection of assessment and treatment components, and to evaluate their responses to 24 statements on a 5-point Likert scale reflecting their alignment with recommendations from Clinical Practice Guidelines. A total of 149 physiotherapists (mean age 39.26 ± 11.50, and gender-balanced) participated, amongst which 35 (23.49%) were classified as ‘adherent’, and 46 subjects (30.87%) were labelled as ‘partially adherent’ to Clinical Practice Guidelines. Participants’ assessment and therapeutic tools selection was generally aligned with the Clinical Practice Guidelines’ extolled recommendations, except for tender points examination and massage therapy. A number of 14 out of the 24 statements exceeded the predefined 70% agreement threshold. The current study reflects an adequate level of knowledge and adherence amongst physiotherapists in Denmark. Further research and targeted implementation strategies are needed to reduce the evidence-to-practice gap.

**Supplementary Information:**

The online version contains supplementary material available at 10.1007/s00296-026-06084-6.

## Background

Fibromyalgia (FM) is a chronic condition characterized by central sensitization and dysregulation of pain processing, manifesting as widespread musculoskeletal pain, fatigue, sleep disturbances, and cognitive dysfunctions [1, 2]. The multifactorial mechanisms surrounding the condition generate a difficult approach to FM, progressively improved by different models, such as the recently introduced FITSS model (Fibromyalgia: Imbalance of Threat and Soothing Systems), which represents an attempt to integrate essential psychosocial and neuropsychological mechanisms in the field of FM, and through which, moreover, constructs as invalidation have been recently identified as a common problem in FM [3]. Despite the fact that new trends and diagnostic criteria are arising and changing the diagnostic paradigm of FM [4], the diagnosis has evolved from a former tender-point model to a more symptom-based and patient-reported framework since, currently, the diagnosis of FM is based on the following clinical findings: (1) generalized pain, (2) duration of symptoms (not explained by other diagnoses) for at least 3 months, (3) widespread pain index (WPI) ≥ 7 and symptom severity scale (SSS) score ≥ 5 points (or WPI of 4–6 points and SSS ≥ 9 points) [5]. Within the specific Danish healthcare framework, FM is clinically conceptualized as part of the Bodily Distress Syndrome (BDS) spectrum, a nationally adopted diagnostic model that unifies functional somatic disorders based on multisystem symptoms rather than organ-specific pathologies [6, 7]. This classification may influence physiotherapists’ diagnostic reasoning and prioritization of treatment modalities, particularly in interdisciplinary settings where BDS is a guiding construct. Epidemiological data from a recent systematic review estimated the FM prevalence in Denmark at 1.8–4.6%, with an important female predominance, in a 3:1 ratio [8]. Notably, over 60% of Danish FM patients present with comorbid anxiety, depression, or overlapping BDS conditions (e.g., irritable bowel syndrome, chronic fatigue, …), reinforcing the clinical utility of this transdiagnostic approach [9–12]. The disorder significantly decreases patients’ quality of life with a substantial impact on patients’ ability to work, participate in social activities, and experience daily enjoyment. It also leads to increased use of welfare benefits, including sickness or unemployment benefits, as well as disability pensions [13–15].

Clinical practice guidelines (CPGs) for the approach of FM currently emphasize a multimodal, patient-centered approach [16–18], more specifically when several profiles exist within the FM population, a fact that points out to the need for specific treatment options given the different profiles [19]. While pharmacological options, such as low-dose amitriptyline, duloxetine, or pregabalin are reserved for refractory cases – reflecting cautious prescribing practices to avoid polypharmacy risks [20] – non-pharmacological interventions, such as patient education, exercise, and cognitive-behavioural therapy, are prioritized as first-line treatments, due to their proven efficacy in reducing pain and fatigue, and improving functional capacity, sleep, and overall quality of life [21–25]. In Denmark, these recommendations align with national healthcare strategies promoting physiotherapy and psychological support, often delivered through multidisciplinary pain structures [13, 26, 27].

Despite the aforementioned, a recent systematic review has shown that clinical practice continues to emphasize the use of antidepressants (67%) while non-pharmacologic therapies remain underutilized [28]: this fact illustrates the existence of persistent gaps between the professionals’ adherence to diagnostic criteria (69%) and the general practitioners’ knowledge (38%). In the UK, solely one out of three healthcare providers implement non-pharmacological interventions for the therapeutic approach of FM [29]. In Saudi Arabia, physiotherapists report a relevant lack of confidence in managing FM [30]. In Spain, our research group has recently highlighted that only 28% of physiotherapists adhere to CPGs for FM [31]. On another note, Italian physiotherapists have demonstrated adequate knowledge and compliance to the referred CPGs, despite the existence of implementation barriers, including limited clinician confidence, self-reported knowledge gaps, and psychosocial complexities in the management of FM [32]. Across all the contexts, common challenges include limited levels of familiarity with CPGs and low confidence in their application. Therefore, whilst international studies highlight widespread implementation challenges among physiotherapists, it remains unclear how these challenges manifest and impact clinical practice. In the specific framework of Denmark’s healthcare system, in which specific structures (e.g., The Research Clinic for Functional Disorders) aim to standardize care [13], understanding the level of physiotherapists’ adherence to FM guidelines could be of paramount importance to enhance care and optimize health-related outcomes among this population.

Thus, the objective of the current study was to assess the level of adherence and knowledge of CPGs among Danish physiotherapists in the management of FM, with the aim of identifying gaps between the current scientific evidence and the actual clinical practice, based on the hypothesis that a certain extent of evidence-to-practice gap may exist, as in previous similar studies conducted in different countries and/or conditions. Findings from this study may guide targeted interventions to strengthen guideline implementation and ultimately improve patient care in Denmark.

## Methods

### Study design

A cross-sectional study was conducted to investigate the level of knowledge and adherence to CPGs on FM among Danish physical therapists. This study adhered to the general recommendations of both the STROBE guidelines for observational studies and the CROSS Checklist for Reporting of Survey Studies [33, 34], in compliance with the ethical standards set by the Declaration of Helsinki and was approved by the Research Committee of the University of Murcia (under the code 4823/2024, approved and certified on February 28th, 2024).

### Participants

The inclusion criteria required participants (i) to be currently practicing as physiotherapists in Denmark, and (ii) to have treated at least one patient with FM within the two years preceding the study onset. Professionals who answered negatively to either one of these criteria received a thank-you message, and they were not allowed to proceed with the survey.

Participants were contacted individually, via their professional email addresses obtained from publicly available contact information of clinical facilities on Denmark’s national health portal (www.sundhed.dk). No specific reminders were sent to participants not having responded to the initial email. Additionally, a link to the questionnaire was shared on social media (LinkedIn), and the authors also encouraged its distribution across different socio-professional networks.

All participants provided their online written and explicit informed consent to participate in the study.

### Procedure

Data were collected by means of an ad-hoc survey developed through Google Forms, in compliance with the General European Data Protection Law [35]. An online survey was used, specifically tailored for this purpose, created by our research group, and previously used in a similar study conducted in Spain [31]. The Danish translation and adaptation of the instrument was performed in accordance with good practice principles published by Wild et al. [36]. Once the instrument translated and adapted, ten professional physiotherapists specialized in musculoskeletal rehabilitation tested the pre-final version for clarity, comprehensibility, and suitability for the target group, therefore confirming its face validity, defined as the extent to which the test is going to measure what it is supposed to measure [37], resulting in a final Danish version, provided as Supplementary Material (in English). On average, the professionals involved in testing the pre-final version took 5 min and 13 s to complete the questionnaire. Confidentiality and anonymity were maintained through the assignment of a code to participants and specifically masking their identity and/or any potential identification of the subject.

Data collection encompassed the period between June 14th, 2024, and March 5th, 2025. Participants initially received, by email, a cover letter, presenting and detailing the main purpose of the study, and then provided the corresponding informed consent.

The survey followed the format and methodology used in the study conducted among Spanish physiotherapists from our research group [31] (which was, in turn, inspired by the instruments previously developed by Battista [38] and Caffini [39]. All three instruments, i.e., ours and those from Battista and Caffini, are licensed under a Creative Commons Attribution 4.0 International License, which permits use, sharing, adaptation, distribution and reproduction as long as the appropriate credit to the original author and the source is provided. The questionnaire was organized into three parts: (i) Sect. 1 (questions 1–12) had a first subsection, focusing on the study details, eligibility criteria, and informed consent, whereas a second subsection collected sociodemographic information; (ii) Sect. 2 (questions 13–15) focused on how professionals would approach a clinical case, through the use of clinical vignettes, which are recognized as valid tools for evaluating both clinical decision-making and adherence to evidence-based practice [40]. The clinical case itself involved a female patient diagnosed with FM, providing socio-demographic and symptom-based data: respondents were asked to select, from a list, the elements that they would consider for the adequate assessment, treatment, and expected duration of the therapeutic program; (iii) in Sect. 3 (questions 16–39), participants were asked to express their level of agreement with 24 statements using a 5-point Likert scale, in a 1 (completely disagree) to 5 (completely agree) range. Study subjects were considered to agree with the statements whenever the score was 4 or 5 and, conversely, to disagree with the statements if the answer scored in a 1-to-3 range [31]. To reduce acquiescence bias, which is the natural tendency to agree with all survey statements [41], seven out of the 24 statements from Sect. 3 were inverted, so that disagreement with these statements indicated agreement with the CPGs. The questions displayed in both Sects. 2 and 3 were based on the EULAR and SIR CPGs for diagnosing and managing fibromyalgia [16, 17].

The reporting standards for designing, conducting and reporting survey studies by Zimba and Gasparyan [42] were followed. Table 1 summarizes the different statements and recommendations from both CPGs.

**Table 1:** Summary of recommendations from clinical practice guidelines

		Assessment	Treatment and Management
Recommendations	For Statements	Assessment of pain, function, comorbidities and psychosocial context. Abidance of laboratory tests and radiographic analysis.	Physical therapies: Aerobic resistance training; Strengthening exercise; Water activity/water jogging; Thermal therapy. Psychological therapies: Behavioral-cognitive therapy; Therapeutic Writing. Non-conventional therapies: Meditative movement therapies (qigong, yoga, tai chi); Mindfulness; Acupuncture; Hydrotherapy. Self-management and promotion of self-efficacy. Patient education. Joining a Fibromyalgia Patient Association.
	Against Statements	Tender points examination.	Biofeedback. Massage. Homeopathy. Chiropractic. Leaving work.
	Controversial Statements Discrepancy between CPGs	**-**	Hypnosis. Guided imagination.
	Neutral Statements Statements not disclosed in the CPGs	Neurological examination. Gait and posture assessment.	Stretching. Postural reeducation. Magnetotherapy. Ultrasounds. Electrotherapy. Trigger points.

*CPGs* Clinical practice guidelines

### Variables

Since the primary objective of this study was to elucidate the level of knowledge and adherence to ultimately ascertain the degree of conformity in relation to the EULAR and SIR guidelines [16, 17], the responses from Sect. 2 were compared to the statements from the EULAR and SIR CPGs (as summarized in Table 1), and participants were therefore classified as “adherent” (whenever all the options marked were indeed recommended -which entails that they could not have marked all the recommended actions, but solely a percentage of the recommended items- and none of the “against statements”), “partially adherent” (choosing recommended options along with “neutral statements”), and “non-adherent” (selecting at least one “against statement”, or either opting not to treat the patient or to treat him/her for less than five sessions) [38]. Controversial statements were excluded because of differing opinions between the two CPGs employed as the standard in the current research.

### Statistical analyses

In Sect. 1, a descriptive analysis was conducted to characterize the sample. Section 2 involved categorizing participants into the “adherent”, “partially adherent”, and “non-adherent” groups. The characteristics from each subgroup were then compared using Chi-Square (or Fisher’s exact) tests and ANOVA tests, according to the qualitative or quantitative nature of the different variables analysed. Focusing on Sect. 3, participants who either fully or partially agreed with a statement (or, conversely, disagreed with the opposite statement) were considered to generally agree with the CPG recommendations. The overall consensus for each statement was assessed, with a ≥ 70% agreement threshold used as a consensus in the absence of a standard benchmark [43].

All analyses were performed using IBM SPSS Statistics for Windows, Version 28.0 (Armonk, NY, USA: IBM Corp; 2016), with a p-value of significance set at *p* < 0.05.

The sample size was calculated using the Cochran’s modified formula for finite populations [44], as of the current population of 13,393 physiotherapists in Denmark provided by World Physiotherapy (https://world.physio/membership/denmark). With a 95% Confidence Interval and 7% margin of error [45], the minimum required sample size was 124 subjects.

## Results

### Section 1-participants’ characteristics

Out of 250 invited physiotherapists, 166 (66.40%) agreed to participate, and 149 (89.76% out of the 166 having accepted participation) met the eligibility criteria. Concerning the 17 professionals excluded, all of them provided concurrent negative responses to the two defined inclusion criteria. The sample included consisted, thus, of 149 physiotherapists with mean age ± standard deviation of 39.26 ± 11.50 years, and gender-balanced (55% female vs. 45% male professionals). Amongst the professionals included, the average years of professional experience corresponded to 12.34 ± 9.72 years, and over 70% of the physiotherapists had an undergraduate academic level of studies. Also, three out of four professionals had previous training in the specific field of FM, whilst roughly half of the sample had previously read a Clinical Practice Guideline in FM. No missing data were reported. Further information on sociodemographic and academic-professional characteristics of the sample is displayed in Table 2.

**Table 2:** Sociodemographic and academic-professional characteristics of the sample

Participants’ characteristics (*n* = 149)	Mean ± Standard Deviation. Frequency (Percentage)
Age (years)	39.26 ± 11.50
Gender (female)	82 (55)
Professional Experience (years)	12.34 ± 9.72
*Academic level of studies*	
Undergraduate Degree	108 (72.50)
Professional Specialization in Specific Area	29 (19.40)
Master’s Degree or PhD	12 (8.10)
Previous training in Fibromyalgia (Yes)	114 (76.50)
Having previously read Clinical Practice Guideline in Fibromyalgia (Yes)	79 (53.00)

### Section 2-adherence levels

**Fig. 1:**
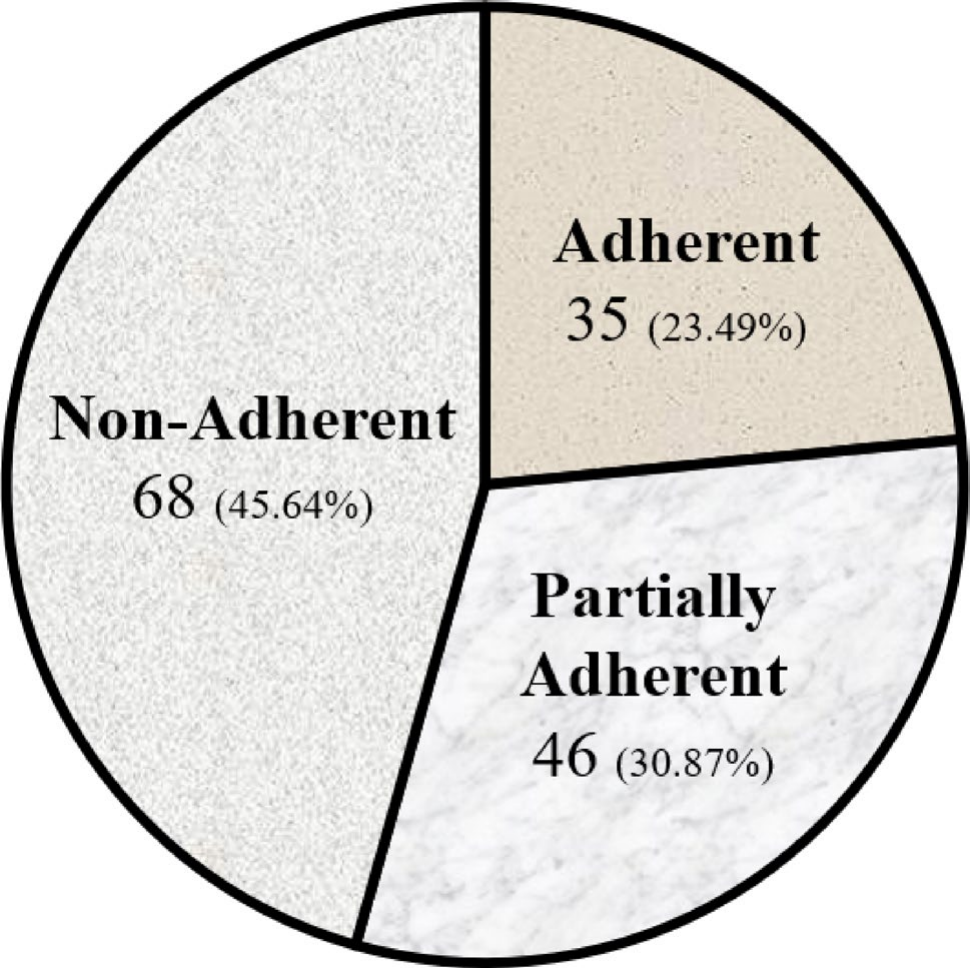
Level of adherence of the study participants

Figure 1 graphically shows the distribution of participants according to their level of adherence. As displayed, a total of 35 professionals (23.49%) reported providing measures included among the CPGs recommended statements and were therefore classified as “adherent”. In the “partially adherent” group (i.e., at least one of the neutral statements was included, either in the assessment or the therapeutic strategy), neurological assessment was supported by 27 physiotherapists, while 15 extolled the assessment of gait and/or posture. As for the treatment, stretching was endorsed by 17 physiotherapists, whereas 5 recommended postural reeducation. Trigger points and electrotherapy were recommended by 3 physiotherapists in both cases. Due to overlapping of some recommendations, the group was composed of 46 physiotherapists (30.87%). A number of 68 physiotherapists (45.64%) were classified as “non-adherent”. Some professionals chose recommendations classified as “against statements” in the CPG, so that there were 55 responses endorsing tender point examination and, in the therapeutic dimension, massage was supported by 24 professionals, chiropractic techniques were extolled by 7 physiotherapists, and 2 professionals supported biofeedback and homeopathy. Moreover, less than 5 sessions of treatment were recommended by 22 physiotherapists.

“Self-management and promotion of self-efficacy” (*n* = 140) was the most endorsed therapeutic statement, followed by “patient education” (*n* = 107), “strength exercises” (*n* = 101), and “aerobic exercise” (*n* = 97). As for the neutral statements, “stretching” was supported by more than one out of four physiotherapists. The most endorsed “against” therapeutic statement was “massage”, accounting for 16.11% of the surveyed sample. Table 3 displays the response rate for each one of the different statements explored. Focusing on the number of sessions potentially set, a total of 34 physiotherapists (22.80%) suggested a number below 5, whilst 91 (61.10%) study subjects chose the 5-to-10 session range, and 24 (16.10%) selected the option corresponding to over 10 sessions of treatment.

**Table 3:** Response rate for each recommendation statement (*n* = 149)

Statement	Type			Responses	
	For	Neutral	Against	*N*	%
*Assessment*					
Assessment of pain, function, comorbidities and psychosocial context	***●***			140	93.96
Tender points examination			***●***	55	36.91
Neurological examination		***●***		51	34.23
Gait and posture assessment		***●***		41	27.52
Abidance of laboratory tests	***●***			36	24.16
Abidance of radiographic analysis	***●***			13	8.72
*Treatment & Management*					
Self-management and promotion of self-efficacy	***●***			140	93.96
Patient education	***●***			107	71.81
Strengthening exercise	***●***			101	67.78
Aerobic resistance training	***●***			97	65.10
Joining a Fibromyalgia Patient Association	***●***			93	62.41
Behavioral-cognitive therapy; Therapeutic writing	***●***			93	62.41
Mindfulness	***●***			69	46.31
Stretching		***●***		43	28.86
Meditative movement therapies (qigong, yoga, tai chi)	***●***			24	16.11
Massage			***●***	24	16.11
Postural reeducation		***●***		14	9.39
Acupuncture	***●***			13	8.72
Trigger points		***●***		10	6.71
Hydrotherapy; Water activity and jogging	***●***			9	6.04
Electrotherapy		***●***		9	6.04
Chiropractic			***●***	7	4.69
Work leave			***●***	4	2.68
Homeopathy			***●***	2	1.34
Biofeedback			***●***	2	1.34
Ultrasounds		***●***		2	1.34
Thermal therapy	***●***			1	0.67
Magnetotherapy		***●***		0	0.00

Concerning the comparisons established across adherence-based groups (i.e., adherent vs. partially adherent vs. non-adherent), solely the fact of having had specific training in FM (Chi-square = 36.468; p-value < 0.001) displayed statistically significant differences across groups.

### Section 3-agreement with CPG recommendations

**Fig. 2:**
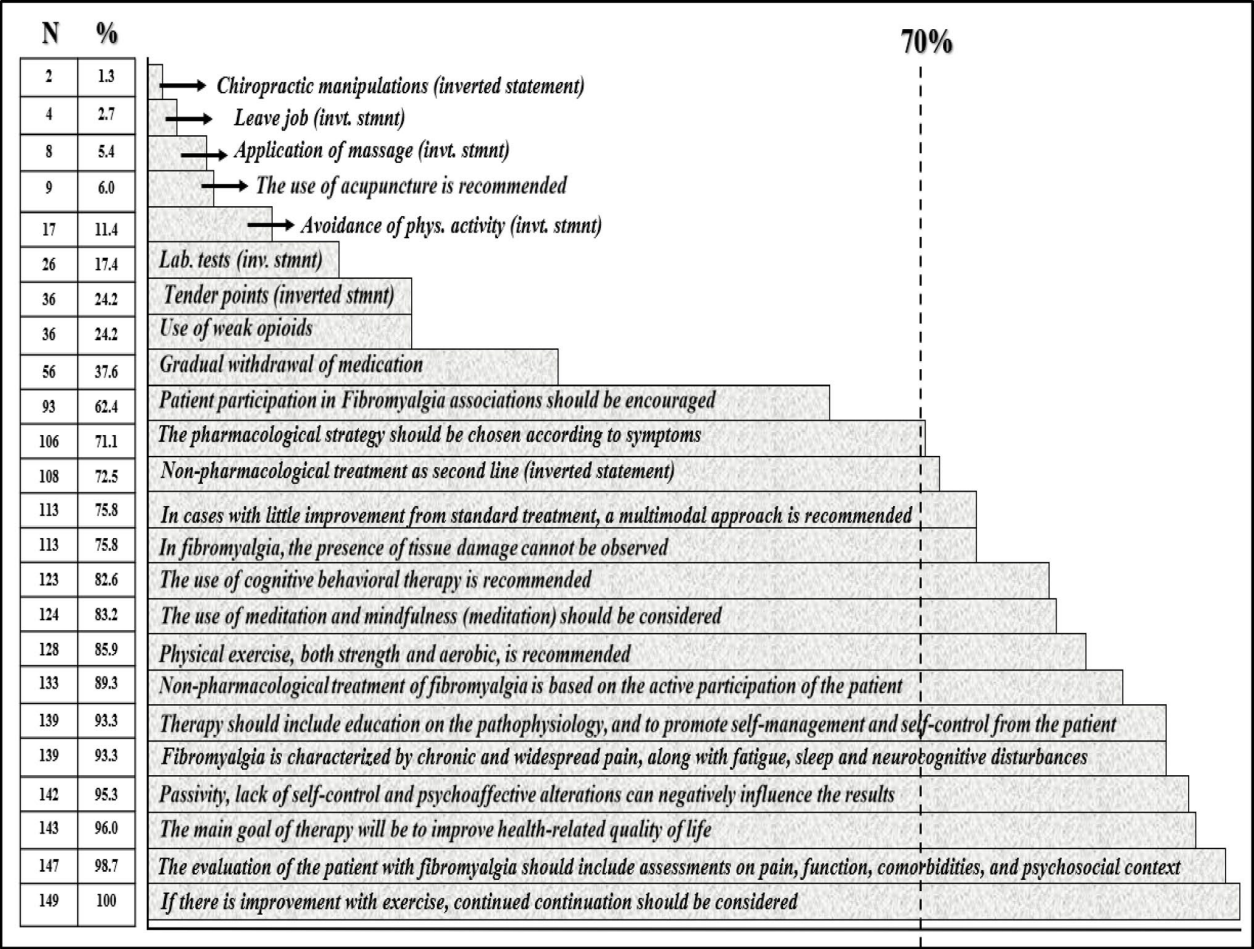
Level of agreement per statement

Figure 2 shows the physiotherapists’ global level of agreement with the CPG recommendations, displaying both the number and the percentage of agreement. The 70% predefined cut-off point generated two different groups: recommendations overtaking an acceptable threshold (14 statements), and those not reaching the aforementioned threshold (10 items, among which six of the inverted statements).

## Discussion

This study aimed to assess the knowledge and adherence to CPGs on FM among Danish physiotherapists. Findings showed that approximately 23% physiotherapists were adherent, 31% partially adherent, and 46% non-adherent. While over half of the participants had prior training in FM and 53% had read a CPG, guideline-consistent decisions were far from universal. These results reflect a persistent ‘evidence-to-practice gap’ in physiotherapy – previously described across healthcare professions [46] – and, moreover, they underscore the need for targeted implementation strategies in the Danish context.

This study highlights that roughly half of the sample had previously read or clinically applied the recommendations of a CPG in the specific field of FM, a fact that aligns with a previous similar study [47], as well as with our previous research conducted amongst physiotherapists in Spain [31]. Moreover, in the current study, having prior training in FM was significantly associated with higher levels of adherence to CGP. This finding highlights that education makes a difference, and it is, furthermore, consistent with previous research amongst Spanish physiotherapists [31]. In contrast, neither the years of professional experience nor the academic level achieved were significantly related to adherence in the Danish sample. Interestingly, while professional experience also showed no effect in the Spanish context, higher academic qualifications did predict better adherence in Spain. These differences may reflect variations in how pain science is integrated into national physiotherapy curricula. The answer to this difference, alongside the fact that the percentage of subjects in the non-adherent group was slightly lower in Denmark than in Spain (45.64% vs. 50.00%, respectively), could lie on the fact that the PT discipline was first implemented in Denmark wide before than in Spain (the Association of Danish Physiotherapists was founded in 1918, whilst the first schools for physiotherapy were created in Spain in 1969) [48], and, therefore, the level of knowledge may therefore be more thoroughly grounded in the Danish framework.

Despite being excluded from current CPGs due to limited diagnostic value [5, 16], tender point examination was selected by 36,91% of participants, making it the second most commonly chosen assessment strategy. Similarly, 16% of respondents endorsed massage therapy, although it is not supported by current guideline evidence due to its limited efficacy in pain outcomes [17]. These findings echo patterns observed in other contexts, including Spain and the UK [29, 31], and confirm the persistence of outdated practices in fibromyalgia management internationally [28]. The continued use of such approaches among Danish physiotherapists may reflect historical training models, a lack of continuing education, or strong patient preferences, underscoring the need for improved guideline dissemination and implementation. As far as the therapeutic approach is concerned, the seven more chosen statements were indeed “for” recommendations, with high percentual figures in self-management and promotion of self-efficacy, patient education, strengthening exercise, aerobic resistance training, joining a fibromyalgia patient association, behavioral-cognitive therapy and therapeutic writing, or mindfulness (in a 46-to-93% range). The importance and relevance of exercise, in all its typologies, as a therapeutic tool of paramount importance in the field of FM has already been confirmed through a significant increase in the production of scientific literature on FM and exercise over the last decade [49].

Several guideline-endorsed interventions, such as acupuncture (6%), hydrotherapy (6%), and thermal therapy (0.7%), were rarely selected by participants. This underutilization may reflect contextual factors within Danish physiotherapy, such as limited access, reimbursement restrictions, or low integration of complementary approaches in standard practice. Similar trends have been observed internationally, especially in terms of the acupunctural perceived effects, in Spain (22.08%) and Saudi-Arabia (below 30%) [30, 31], suggesting that even evidence-supported modalities may be underused due to practical, systemic, or cultural barriers rather than to a lack of knowledge. Acupuncture, moreover, was the lowest non-inverted statement to be chosen by the surveyed sample (solely nine subjects, i.e., 6% of the sample), in a coherent and consistent line with the low percentage of selection within the therapeutic tools suggested. Other three non-inverted statements did not reach the threshold for agreement of 70%: two of them specifically focused on medication (opioids and medical deprescription), whilst the extant one corresponded to enrolling FM associations. While the medication-based statements may be explained by the fact that physiotherapists do not prescribe medication in the framework of the Danish Healthcare System [50], the low figures stated concerning the recommendation of joining patient associations is perfectly coherent, in terms of frequency and percentage, with the number of responses concerning the “for” recommendations, therefore suggesting that the 93 (i.e., 62.4%) positive responses on this statement correspond to the same 93 subjects having endorsed and supported its use as a therapeutic tool of paramount importance within the selected strategies or recommendations. This fact somehow clashes with the higher 78% of agreement with the statement found in Spain, and may reveal, in turn, difficulties in the general knowledge and purposes of patients’ associations not only from patients, but also from their family carers and professionals, therefore hindering the possibility to find, navigate and use the complex systems of support, despite the existence of several care pathways, as already explored and concluded for other chronic conditions [51].

The results exposed shall nonetheless be interpreted in the light of the study’s methodological limitations. Most notably, the criterion adopted for both eligibility of the physiotherapists and the adherence-based classification was restrictive in both cases: the fact that one of the potential exclusion criteria corresponded to subjects not having treated at least one patient with FM in the last two years aimed to retain professionals with a recent approach to FM but it may have excluded to those professionals having had a thorough theoretical and academic training on the condition with little therapeutic experience. It may have excluded competent professionals with theoretical knowledge but limited recent clinical exposure. Moreover, the recruitment process may have included a potential representation bias of public hired physiotherapists and physiotherapists at private clinical practice, a fact that shall be considered and potentially controlled in further research. Also, the recruitment strategy relied on social media dissemination and voluntary participation, a fact that may have favored the inclusion of physiotherapists specially motivated and/or engaged. This introduces a potential selection bias that could limit the representativeness of the sample. Despite the fact that voluntary participation is cost-effective and allows a wide geographic reach, some potential biases, as non-response bias, have been specifically linked to this recruitment strategy [52]. Further studies shall focus on implementing random sampling techniques or using post-stratification techniques, to avoid this potential bias ultimately affecting the generalizability of the findings.

On another note, the criterion for classification as “non-adherent” was based on previous research, but a professional having chosen all the “for” options and having solely marked one “against” recommendation would have been automatically labelled as “non-adherent”. Despite appearing as strict criteria, we adopted this operational definition of adherence based on previous works in the field of assessing adherence to CPGs amongst PTs in different conditions [31, 38]. Finally, the current study did not control the potential professional-based social desirability response bias, even though efforts were channeled with the introduction of seven inverted statements to ensure acquiescence bias, a fact that could have also minimized desirability response bias.

Our results underscore and evince the existence of barriers to guideline uptake, an aspect in which several authors have already put the focus across different health strata and clinical approaches: these barriers for the implementation of guidelines therefore involve different levels, not only in the healthcare system, but also on organizational, societal and cultural specificities, and also including individual attitudes. The aforementioned factors altogether should be addressed with specific and tailored policy-driven strategies [53, 54].

In summary, this study highlights both promising adherence patterns and persistent gaps in evidence-based practice among Danish physiotherapists treating FM. Targeted educational strategies, better dissemination of guidelines, and structural support may enhance physiotherapists’ capacity to deliver optimal care for this complex patient group. Future work should explore targeted and context-sensitive implementation strategies to equip physiotherapists with the knowledge, confidence, and structural support to bridge the guideline-practice gap in fibromyalgia care.

## Supplementary Information

Below is the link to the electronic supplementary material.

Supplementary Material 1

## Data Availability

The datasets used and analyzed during the current study are available from the corresponding author on reasonable request.
