# Supplementary material for: Clinical practice guidelines in fibromyalgia. Physiotherapists’ adherence in Denmark: a cross-sectional web-based survey study
Source: Rheumatol Int. 2026 Feb 18;46(3):49. doi: 10.1007/s00296-026-06084-6 (PMC12917084; doi:10.1007/s00296-026-06084-6)
Supplement: Supplementary file 1 — Supplementary Material 1 [file 296_2026_6084_MOESM1_ESM.docx]

**Supplementary File “X”**

**Survey in English language**

**Physiotherapists’ Adherence to Clinical Practice Guidelines in Fibromyalgia: A Cross-Sectional Survey**

Dear participant, you have been invited to take part in the study titled “Physiotherapists’ Adherence to Clinical Practice Guidelines in Fibromyalgia: A Cross-Sectional Survey.”

Before completing the survey, it is important that you understand the reason for the study and what you will be asked. The first section includes information about the study and informed consent in addition to the demographic characteristics of the participants. In the second section, you will be presented with two clinical cases on which you will have to mark which treatments you would perform. In the third section, you will have to indicate your level of agreement with different statements about the management of fibromyalgia.

In case of doubt or query, the researchers are at your complete disposal. The person to whom you should contact is Katrine Oerte Frederiksen, lecturer at the physiotherapist training at UC SYD, on email kofr@ucsyd.dk

Finally, thank you for your interest and your important participation.

**Section 1: demographic characteristics of participants**

A. Information about the research and informed consent

1. I declare that I have read and understood the “study information” file (add link, file or, if via Google Form, attach screenshot). Yes/No.
2. I declare that I have read and understood the file “information on the processing of personal data” and I declare that I give my consent to the University of Murcia to use my personal data for the purpose described in the document (add link, file or, if by Application Form). Google, attach screenshot). Yes/No.

B. Demographic characteristics of participants

1. Birthdate: ________________.
2. What is your gender identity? Female/Male/Transgender/Non-Binary/Other/Prefer not to answer.
3. Do you have a degree in Physiotherapy from a Danish University? Yes/No.
4. ¿In what year? _______________.
5. Higher level of training (indicate the highest):
   1. Diploma/graduate in Physiotherapy
   2. Unofficial postgraduate/master's degree
   3. Official master's degree
   4. PhD
   5. Other (specify):
6. Years of professional experience: __________________.
7. Do you currently work as a physiotherapist in Denmark? Yes (inclusion)/No (exclusion).
8. In which community? __________________
9. In what area do you carry out your main activity? (to which I dedicate the most hours)
   1. Public area
   2. Private area
   3. Mutual.
   4. Education/research
10. Have you treated any patients with fibromyalgia in the last two years? Yes (inclusion) / No (exclusion).
11. Have you completed any training related to fibromyalgia or that included fibromyalgia management? Yes/No.
12. If yes, please indicate the number of hours:
    1. Less than 20
    2. 20-40
    3. 41-60
    4. More than 60
13. Have you read any clinical practice guidelines on fibromyalgia? Yes/No.

**Section 2: Clinical case (adherence research)**

Please read the following clinical case carefully and indicate below how you would approach the indicated patient.

A 59-year-old woman, diagnosed with fibromyalgia, who lives with her husband and two children, ages 27 and 25. He is slightly overweight (BMI 26.4). He has been reporting generalized pain for years. She says that these pains vary in intensity over time. She also reports that she feels more tired than usual (she has to take breaks when carrying out her daily activities), that her sleep is not restful, and that she has gastrointestinal discomfort. diffuse. He has worked as a salesperson for 20 years. He says that, although his job causes him stress and anxiety, he handles it well.

A. Indicate what aspects you would include in the assessment. Mark as many as you think is appropriate.

- “Tender points”
- Pain
- Neurological examination
- Function
- Posture and gait exploration
- Comorbidities
- Lab tests
- Psychosocial context
- Radiological images

B. Indicate what treatment techniques you would include or what recommendations you would give to the patient. Check as many as you think is appropriate.

- Instructions for self-management
- Educate the patient about her pathology
- Promote self-efficacy
- Strength exercise
- Aerobic exercise
- Stretching
- Acupuncture
- Postural correction
- Massage therapy
- Chiropractic
- Hydrotherapy-hydrokinesitherapy.
- Balneotherapy-thermal therapy
- Magnetotherapy
- Ultrasound
- TENS
- Guided imagery
- Recommend fibromyalgia association
- Cognitive behavioral therapy
- Homeopathy
- Biofeedback
- Mindfulness (meditation)
- Yoga-Tai Chi
- Treatment of trigger points (inhibitory pressure, dry needling, etc.)
- Leave the job

C. Indicate how many treatment sessions you would perform (in total).

- <5
- 5-10
- >10

**Section 3: Consensus on statements (knowledge research)**

Please read the following statements carefully and honestly mark your level of agreement with each of them from 1 (completely disagree) to 5 (completely agree). Check only one option per statement. Please, mark the answer that best reflects your first reaction; do not spend too much time on each question.

| **Statement** | **1.**  **Completely disagree** | **2.**  **Partially disagree** | **3.**  **Neither agree nor disagree** | **4.**  **Partially agree** | **5.**  **Completely agree** |
| --- | --- | --- | --- | --- | --- |
| 1. Fibromyalgia is a pathology characterized by chronic and widespread pain, along with other symptoms such as fatigue, sleep and neurocognitive disturbances. |  |  |  |  |  |
| 1. In fibromyalgia, the presence of tissue damage cannot be observed. |  |  |  |  |  |
| 1. The evaluation of the patient with fibromyalgia should include assessments on pain, function, comorbidities, and psychosocial context. |  |  |  |  |  |
| 1. In general, laboratory and imaging tests will be necessary to reach a diagnosis of fibromyalgia. **(inverted statement)**. |  |  |  |  |  |
| 1. Palpation of the “tender points” is relevant in the diagnosis of fibromyalgia as well as in monitoring the evolution. **(inverted statement)**. |  |  |  |  |  |
| 1. The main goal of therapy will be to improve health-related quality of life. |  |  |  |  |  |
| 1. The pharmacological strategy should be chosen according to symptoms and paying attention to adverse effects. |  |  |  |  |  |
| 1. In cases with little improvement from the standard treatment, it is recommended to carry out a multimodal approach (physical activity combined with, at least, psychotherapy) taking the patient's preferences into account. |  |  |  |  |  |
| 1. Weak opioids (tramadol), anticonvulsants/antiepileptics (pregabalin), serotonin and norepinephrine reuptake inhibitors (fluoxetine, paroxetine and duloxetine), as well as tricyclic antidepressants (amitriptyline) and cyclobezaprine and cannaboids can be used to modulate pain. |  |  |  |  |  |
| 1. If there is a positive effect of the medication, its gradual withdrawal should be considered. |  |  |  |  |  |
| 1. The non-pharmacological treatment of fibromyalgia constitutes the second line of action. (**inverted statement)**. |  |  |  |  |  |
| 1. Non-pharmacological treatment of fibromyalgia is based on the active participation of the patient. |  |  |  |  |  |
| 1. Patient participation in Fibromyalgia associations should be encouraged. |  |  |  |  |  |
| 1. Physical exercise, both strength and aerobic, is recommended. |  |  |  |  |  |
| 1. If there is improvement with exercise, continued continuation should be considered. |  |  |  |  |  |
| 1. The rehabilitation program should include the application of massage. **(inverted statement)**. |  |  |  |  |  |
| 1. The use of acupuncture is recommended. |  |  |  |  |  |
| 1. Therapy should include education on the pathophysiology of the disease, in addition to promoting self-management and self-control from the patient. |  |  |  |  |  |
| 1. In acute phases we must recommend rest, avoiding physical activity. **(inverted statement).** |  |  |  |  |  |
| 1. The use of meditation and mindfulness (meditation) should be considered. |  |  |  |  |  |
| 1. The use of cognitive behavioral therapy is recommended. |  |  |  |  |  |
| 1. The therapeutic management should include chiropractic manipulations **(inverted statement)**. |  |  |  |  |  |
| 1. Factors such as passivity, lack of self-control and psychoaffective alterations can negatively influence the results. |  |  |  |  |  |
| 1. The patient with fibromyalgia will be recommended to leave his job (if he/she is active). **(inverted statement)**. |  |  |  |  |  |

**Section 4: Proposal**

Use this question to add any questions, suggestions or comments you deem necessary. Thank you.
